# Genomic correlates of glatiramer acetate adverse cardiovascular effects lead to a novel locus mediating coronary risk

**DOI:** 10.1371/journal.pone.0182999

**Published:** 2017-08-22

**Authors:** Ingrid Brænne, Lingyao Zeng, Christina Willenborg, Vinicius Tragante, Thorsten Kessler, CARDIoGRAM Consortium, CARDIoGRAMplusC4D Consortium, Cristen J. Willer, Markku Laakso, Lars Wallentin, Paul W. Franks, Veikko Salomaa, Abbas Dehghan, Thomas Meitinger, Nilesh J. Samani, Folkert W. Asselbergs, Jeanette Erdmann, Heribert Schunkert

**Affiliations:** 1 Institute for Cardiogenetics, University of Lübeck, Lübeck, Germany; 2 DZHK (German Research Center for Cardiovascular Research), partner site Hamburg/Lübeck/Kiel, Lübeck, Germany; 3 University Heart Center Lübeck, Lübeck, Germany; 4 Deutsches Herzzentrum München, Technische Universität München, München, Germany; 5 Department of Cardiology, Division Heart and Lungs, UMC Utrecht, Utrecht, The Netherlands; 6 University of Michigan, Dept of Biostatistics, 1415 Washington Hts, Ann Arbor, MI, United States of America; 7 Institute of Clinical Medicine, Internal Medicine, University of Eastern Finland and Kuopio University Hospital, Kuopio, Finland; 8 Uppsala Clinical Research Center, Uppsala Science Park, MTC, Uppsala, Sweden; 9 Genetic and Molecular Epidemiology Unit, Department of Clinical Sciences, Lund University, Skåne University Hospital Malmö, Malmö, Sweden; 10 THL-National Institute for Health and Welfare, POB 30, Mannerheimintie 166, Helsinki, Finland; 11 Department of Epidemiology, Erasmus University Medical Center, CA Rotterdam, The Netherlands; 12 Institute of Human Genetics, Helmholtz Zentrum München, German Research Center for Environmental Health, Neuherberg, Germany; 13 DZHK (German Center for Cardiovascular Research), partner site Munich Heart Alliance, Munich, Germany; 14 Institute of Human Genetics, Technische Universität München, Munich, Germany; 15 Department of Cardiovascular Sciences University of Leicester and NIHR Leicester Cardiovascular Biomedical Research Unit, Glenfield Hospital, Leicester, United Kingdom; 16 Durrer Center for Cardiovascular Research,Netherlands Heart Institute, Utrecht, the Netherlands; 17 Institute of Cardiovascular Science, faculty of Population Health Sciences, University College London, London, United Kingdom; Centro Cardiologico Monzino, ITALY

## Abstract

Glatiramer acetate is used therapeutically in multiple sclerosis but also known for adverse effects including elevated coronary artery disease (CAD) risk. The mechanisms underlying the cardiovascular side effects of the medication are unclear. Here, we made use of the chromosomal variation in the genes that are known to be affected by glatiramer treatment. Focusing on genes and gene products reported by drug-gene interaction database to interact with glatiramer acetate we explored a large meta-analysis on CAD genome-wide association studies aiming firstly, to investigate whether variants in these genes also affect cardiovascular risk and secondly, to identify new CAD risk genes. We traced association signals in a 200-kb region around genomic positions of genes interacting with glatiramer in up to 60 801 CAD cases and 123 504 controls. We validated the identified association in additional 21 934 CAD cases and 76 087 controls. We identified three new CAD risk alleles within the TGFB1 region on chromosome 19 that independently affect CAD risk. The lead SNP rs12459996 was genome-wide significantly associated with CAD in the extended meta-analysis (odds ratio 1.09, p = 1.58×10^−12^). The other two SNPs at the locus were not in linkage disequilibrium with the lead SNP and by a conditional analysis showed p-values of 4.05 × 10^−10^ and 2.21 × 10^−6^. Thus, studying genes reported to interact with glatiramer acetate we identified genetic variants that concordantly with the drug increase the risk of CAD. Of these, TGFB1 displayed signal for association. Indeed, the gene has been associated with CAD previously in both in vivo and in vitro studies. Here we establish genome-wide significant association with CAD in large human samples.

## Introduction

Glatiramer acetate (GA), also known under the trade name Copaxone®, is an immunomodulator used in the treatment of relapsing-remitting multiple sclerosis (MS). It is a synthetic peptide consisting of four amino acids[1, 2]. GA is assumed to bind major histocompatibility complex (MHC) molecules and compete with various myelin antigens[2]. This competitive binding affects the presentation of myelin antigens to T-cells. In addition, GA potentially promotes suppressor T-cells[1, 3]. A further mechanism of action is that GA-induced T helper cells secrete high amounts of cytokines such as IL-4/10 and TGF-β[2]. In mice, GA is reported to induce transforming growth factor β 1 (TGFB1) in several cell types such as monocytes[4], Th2/3 cells[5] and brain[6]. In addition, it is known that polymorphisms in the TGFB1 gene alter the GA treatment response[7].

According to the FDA,drugs.com and the copaxone own webpage (copaxone.com), GA is reported to induce hypertension and increase the risk of coronary artery disease (CAD) and myocardial infarction. The exact mechanisms that explain the increased risk of CAD or hypertension under GA treatment are, however, not fully understood.

Understanding the genetic mechanisms underlying a disease can facilitate the identification of new drug targets. Indeed, several drugs have been developed based on genetic findings [8–10]. Moreover, if we know which genes or pathways are targeted by a drug, we may also predict adverse effects based on variants in these genes modulating risk[11, 12]. Here, we reversed this approach to identify new disease risk genes. We screened genes that are reported to interact with a drug that increases the risk of CAD to identify new CAD risk alleles. In a previous study, we identified new CAD risk genes by studying the pleiotropic effects of cyclooxygenase 2 inhibitors[13].

In this work, we first identified genes and gene products reported to interact with GA. These genes were then screened for association in the largest meta-analysis on CAD genome-wide association studies (GWAS), CARDIoGRAMplusC4D 1000G[14]. The underlying idea is that single nucleotide polymorphisms (SNPs) in *cis* with these genes may affect expression or structure of these genes in a similar way as the drug. Hence, we expect that some of these SNPs also may increase the risk of CAD, even though the effect size might vary between drug and variant.

## Methods

The first step of the analysis was to identify genes or gene products reported to interact with GA. For this, we used the Drug Gene Interaction Database (DGIdb)[15]. Second, in a large CAD GWAS dataset, we identified all SNPs within 200kb surrounding the four genes identified in the first step of this analysis. The 200kb window was selected to also include regulatory SNPs affecting gene expression.

### GWAS dataset

The CARDIoGRAMplusC4D 1000Genomes meta-analysis data set consists of 47 GWAS studies including 60 801 CAD cases and 123 504 controls. Ethical approval was obtained from the appropriate ethics committees and informed consent was obtained from all participants. Specifically, the studies involving genome-wide SNP analysis for CAD were approved by the ethics commissions of the University of Regensburg (02/042), the University of Lübeck (04/041) und the Technical University of Munich (406/15s). The GWAS are imputed with the December 2012 1000Genomes phase I integrated haplotypes (ftp://ftp.1000genomes.ebi.ac.uk/vol1/ftp/release/20110521/) (for Methods see Nikpay et al.[14]).

We validated and later combined the CARDIoGRAMplusC4D 1000Genomes meta-analysis data set[14] with GWAS data from CHARGE, deCODE CAD, DILGOM, EPIC, FRISC II GLACIER, METISM, MORGAM FIN, MORGAM FRA, MORGAM GER, MORGAM ITA, MORGAM UNK, PMB, PopGen, SCARF SHEEP, and STR that have been previously reported in references Schunkert et al. [16] and/or Deloukas et at. [17]. Moreover, we included data from GWAS not reported before, i.e. German MI Family Studies V. See detailed information in S1 Table. In total, this combined dataset consists of 82 735 cases and 199 591 controls. Compared to the CARDIoGRAMplusC4D study, we increase the sample size by 21.934 cases and 76.087 controls.

### Meta-analysis

Logistic regression, assuming an additive model, was performed on all single study data. All analyses were adjusted for sex and age. Age was defined as the recruitment age for controls and the event age for cases. We used the fixed-effect inverse variance-weighted meta-analysis to combine single analyses data. Quality control was performed at individual sites and centrally to assure standardized data formats previously agreed criteria including check of consistency of the given alleles across all studies, quality of the imputation, deviation from Hardy-Weinberg equilibrium and call rate. If individuals or single studies did not pass quality control, they were excluded. SNPs were also excluded from the meta-analysis if present in less than 17 GWAS.

For the meta-analysis, we calculated an 'inverse variance weighting'- fixed-effects and a random effects model[18], depending on the heterogeneity between the studies. For heterogeneity calculation, Cochran’s Q was used. The threshold for heterogeneity was phet<0.01. For the combination of the stages (stage1: results of 1000G meta-analysis; stage 2: replication in CARDIoGRAM, CARDIoGRAMplusC4D, GerMIFS V) an 'inverse variance weighting'- the fixed-effects model was calculated and the combined effects and p-values were reported. In total, we evaluated the genomic data from 82 735 cases and 199 591 controls.

### Statistical methods

The number of SNPs tested in the initial screen (for the four genes) is 20,027. We corrected the p-value threshold based on the number of SNPs tested using the Bonferroni correction. Hence, all SNPs with p-values below 2.5x10^-6^ were considered significant.

### Identification of TGFB1 sub-loci

We used Haploreg version 4.1[19] with the European 1000G Phase 1 database for LD calculation. We identified LD blocks based on LD > 0.4 to the lead SNPs. In detail, we repeated the following three steps until no sub-loci with a p-value below 1×10^−6^ were found.

1. Identification of current lead SNP (SNP with the lowest p-value).
2. Identify all SNPs in LD (r^2^>0.4) with the current lead SNP.
3. Remove lead and LD SNPs from dataset

To test for independence between the identified sub-loci, we performed conditional analysis using summary statistic data with the GCTA tool[20]. As reference data used the GerMIFs II study. For conditional analysis, we used the SNPs identified in the above-described sub-loci analysis. We first performed a joint analysis using the–cojo-joint option and then performed the conditional analysis (—cojo-cond) based on the independent SNPs identified in the joint analysis step.

### Functional annotation of SNPs and TGFB1

To evaluate the functional implication of the SNPs, we identified all SNPs in high LD (r^2^>0.8) with the locus lead SNP using the HaploReg version 4.1 database[19]. To estimate the effect of a SNP on gene expression, we identified expression quantitative trait loci (eQTLs) using the publicly available data from Westra et al.[21], GTeX[22] as well as over 100 studies included in the Genome-Wide Repository of Associations between SNPs and Phenotypes (GRASP) database[23]. In addition, we used HaploReg and RegulomeDB[24] to functionally annotate SNPs and performed a literature search for gene functions using Pubmed.

## Results

**Fig 1:**
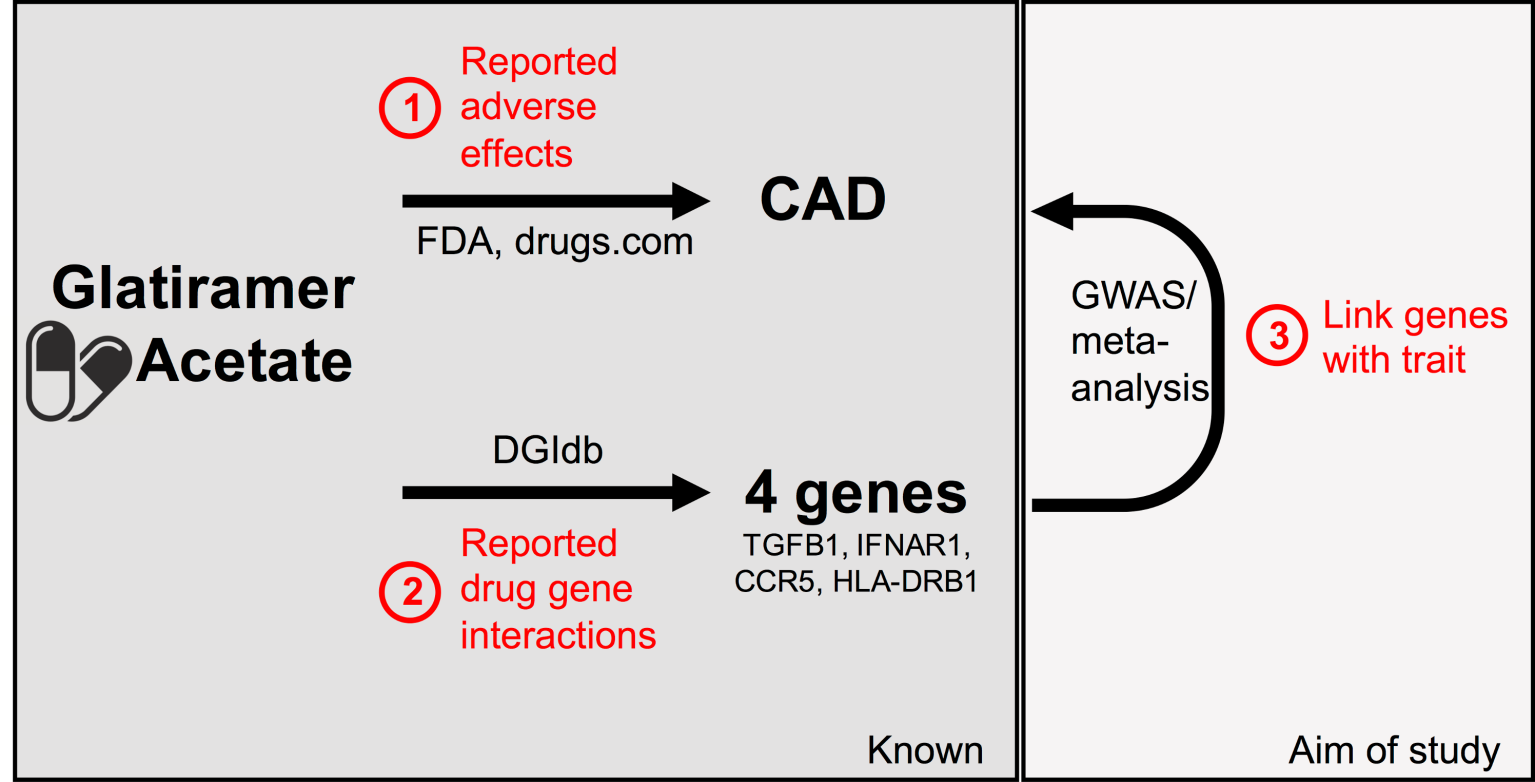
Schematic approach. 1) Identification of reported adverse effect of GA 2) Identify genes reported to interact with GA. 3) Establish a link between the genes identified in 2. and the adverse effect identified in 1).

**Fig 2:**
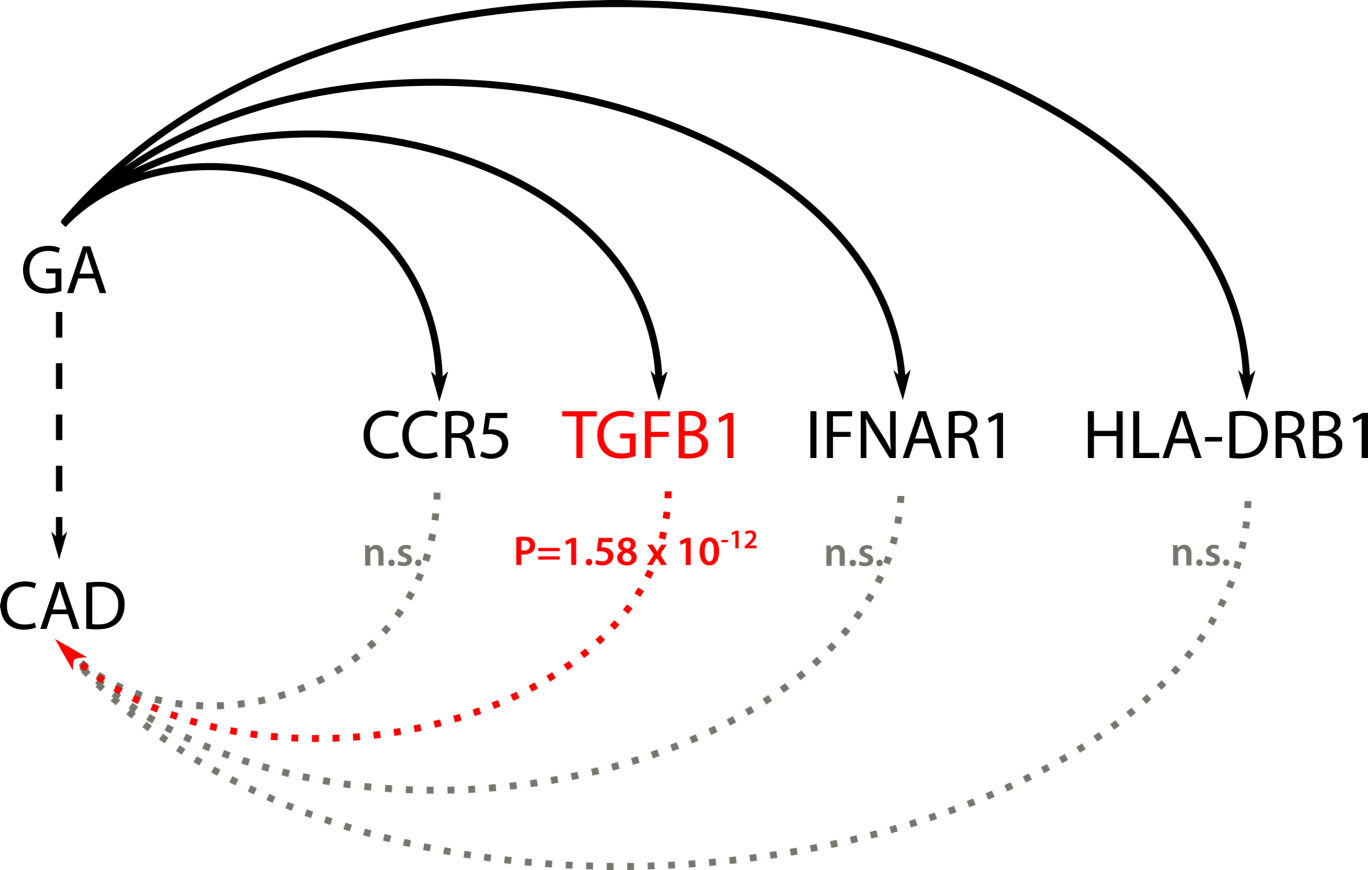
GA interacts with CCR, TGFB1, IFNAR1 and HLA-DRB1 (solid line). Moreover, it is known that GA affects CAD (Coronary Artery Disease) risk (dashed line). In this work, we searched for SNPs associated with CAD in the gene regions representing the GA off target effects (dotted lines). We found a genome-wide significant association for the TGFB1 locus with a p-value of 1.58 × 10^−12^ (red dotted line). n.s.: non-significant; TGFB1: Transforming Growth Factor, Beta 1; CCR5: Chemokine (C-C Motif) Receptor 5 (Gene/Pseudogene); IFNAR1: Interferon (Alpha, Beta And Omega) Receptor 1; HLA-DRB1: Major Histocompatibility Complex, Class II, DR Beta 1.

The principle idea of this approach is illustrated in Fig 1. Using DGIdb[15], we identified four genes reported to interact with GA; CCR5, HLA-DRB1, IFNAR1, and TGFB1. Of these, only the TGFB1 region displayed signals suggesting an association with CAD risk (Fig 2). We validated the GA-TGFB1 interaction performing a literature search[2, 4–6]. Moreover, from a mechanistic point of view, TGFB1 is the only gene with evidence of a functional association with CAD (see S1 Fig). Hence, we did not examine the other genes further.

**Fig 3:**
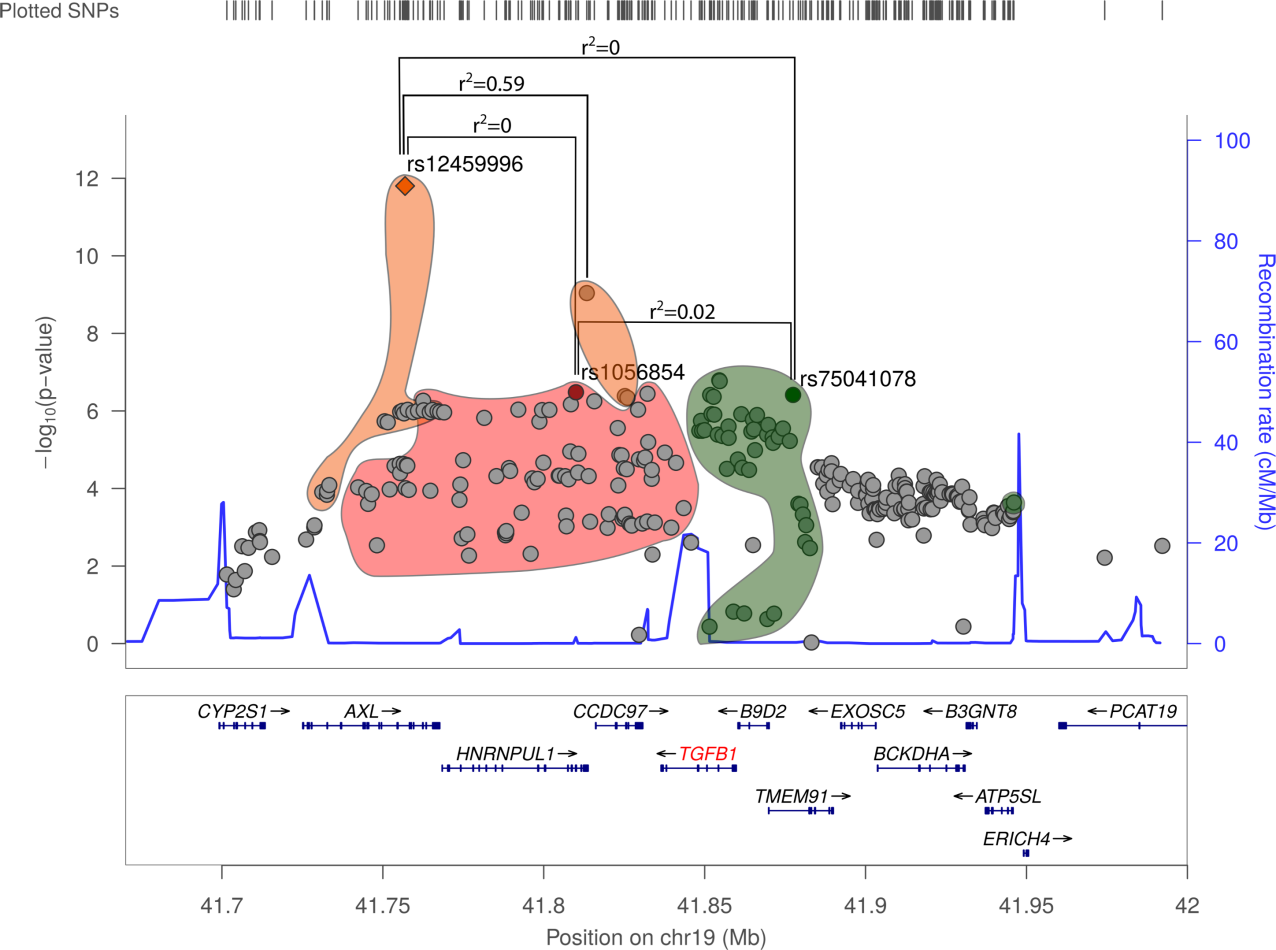
Association sub-loci signal for the TGFB1 locus. The three lead SNPs are shown with the corresponding high-LD blocks (SNPs within r2>0.2.) depicted in orange, red and green. Independent sub-loci were identified with the GCTA conditional analysis tool (see methods). The LD between the lead SNPs indicated and under r^2^<0.1. The three individual LocusZoom plots are found in the S2 Fig.

In our first GWAS look-up, the lead SNP, rs15052, close to TGFB1 yielded a p-value of 2.21 × 10^−7^. In a replication study with independent samples, rs15052 showed a p-value of 9.97 × 10^−4^, hence validating the initial finding. We next combined the data sets and reran the analysis. In this combined meta-analysis, rs15052 yielded a genome-wide significant p-value of 9.11 × 10^−10^. The new lead SNP in the joint meta-analysis, rs12459996 had a p-value of 1.58 × 10^−12^ and an OR of 1.09 (see Fig 3).

The TGFB1 locus presumably harbors several independent sub-loci (see Table 1). Using the GCTA conditional analysis on summary statistic data, we identified three such independent signals. In addition to the genome-wide significant lead SNP, two sub-loci show significant p-values (rs1056854: 3.30 × 10^−7^ and rs75041078: 3.87 × 10^−7^). We conditioned the two new SNPs on rs12459996 in a stepwise approach. rs1056854 showed the lowest p-value after conditioning with rs12459996 with a p-value of 4.07x10^-10^ and an OR of 1.07. Conditioning rs75041078 on the lead SNPs rs12459996 and rs1056854, we get a p-value for the third SNP of 2.21x10^-6^ with an OR of 1.05.

**Table 1:** TGFB1 locus.

Sub-locus	Lead SNP	CAD risk allele	Frequency risk allele	OR (CI) risk allele	P-value combined analysis	P-value joint analysis GCTA	OR risk allele joint analysis GCTA	SNP effect on TGFB1	Regulatory function Haploreg	Regulatory function Regulome score
I	rs12459996	T	0.10	1.09 (1.06–1.12)	1.58x10^-12^	2.73x10^-9^	1.09	Increased expression	enhancer	6
II	rs75041078	A	0.25	1.06 (1.04–1.09)	3.87x10^-7^	4.05x10^-10^	1.07	n/a	enhancer	n/a
III	rs1056854	A	0.86	1.06 (1.04–1.08)	3.30x10^-7^	2.21x10^-6^	1.05	increased expression	enhancer	5

To evaluate the functional implication of the three potentially independent associations, we performed an in-silico evaluation of the lead SNPs and the SNPs in high LD with these (r^2^ > 0.8) (see S2 Table for detailed results).

The genome-wide significant lead SNP rs12459996 (p = 1.58 × 10^−12^; OR = 1.09), is found in a regulatory region and is marked as a strong promoter and enhancer in several cell types including smooth muscle and T-helper cells. As the promoter marks are downstream TGFB1, it is more likely that the regulatory effect on TGFB1 is acting through the enhancer. The lead SNP and SNPs in high LD (r^2^>0.8) are reported to have an eQTL effect on TGFB1 in the thyroid gland (p = 3.0 × 10^−8^, OR = 1.58) and the skeletal muscle (p = 5.6 × 10^−6^, 1.36) (GTEX). The CAD risk allele T is associated with increased TGFB1 expression in both cell types.

The second sub-locus has the lead SNP rs75041078 (conditional p = 4.07x10^-10^, conditional OR = 1.07). This SNP is not in high LD with any other SNP (r^2^>0.8). It is located in the intron of TMEM91 and lies in an enhancer histone mark in neutrophils. We did not find any eQTL effect in the databases searched.

The third sub-locus, with the lead SNP rs1056854 (conditional p = 2.21x10^-6^, conditional OR = 1.05), is also located in a regulatory region, which includes a promoter histone mark in various cell types, including H1-hESC cells. The locus is, however, downstream or rather at the 3′ end of the TGFB1 gene and hence the promoter unlikely to affect the TGFB1 gene. SNPs in high LD are also found in enhancer histone marks in multiple cell types, including T cells, T-helper cells and monocytes. Hence, the locus might have a regulatory effect on TGFB1. Supporting this hypothesis, we identified an eQTL effect on TGFB1 in the adrenal gland, suggesting a link between the SNP and the gene also in other tissues. The CAD risk allele A is associated with increased expression of TGFB1 (p = 1.5*10^−6^, OR = 2.2).

## Discussion

Exploring drug-gene interactions, we identified four genes or gene products to be affected by glatiramer acetate, a drug known to increase the risk of severe coronary events. Analyzing the genomic loci harboring these genes, we identified TGFB1 as a new genome-wide significant locus displaying association for CAD. The results of this analysis give rise to the hypothesis that the known interaction of GA with TGFB1 may be responsible for modulating the risk of CAD. Indeed, the results point towards a novel mechanism for the increased risk of CAD under GA treatment.

The pharmacologic mechanisms of GA in the treatment of multiple sclerosis are not fully understood. The general assumption is that the immune-modulatory activity of GA is related to the change of the T-cell antigen reactivity. Through its presumed binding to the MHC class II, GA is thought to alter the presentation of myelin antigens to auto-reactive T-cells and thereby affects the activity of the antigen presenting cells[25]. It is also known that GA induces the secretion of cytokines such as IL-4/10 and TGF-β in T-helper cells[2], which according to the present data may affect the risk of CAD under GA treatment.

TGFB1 (transforming growth factor, beta1) is a multifunctional peptide controlling multiple cellular functions such as proliferation and differentiation in several cell types. It plays an important role in the pathophysiology of the endothelial and vascular smooth muscle cell. TGFB1 is a very likely CAD candidate gene and has been linked to a range of cardiovascular traits such atherosclerosis, hypertension, inflammation and aneurysm [26–29]. TGFB1 serum levels are also reported to be higher in CAD patients[30]. In addition, several variants within other genes in the TGFB–SMAD signaling pathway have been associated with CAD[26, 29, 31–34]. It is, hence, also possible, that GAs interaction with TGFB1 influences other reported adverse effects such as the increased risk of hypertension. We cannot exclude that the GA related risk of CAD is secondary to GA induced hypertension, but at the same time we can also not exclude other pathways. TGFB1 is involved in multiple cellular functions suggesting increased CAD risk through multiple pathways.

Here, we identified three independent CAD associated signals within the TGFB1 locus. The TGFB1 locus has not been genome-wide significantly associated with CAD before, most likely due to small effect sizes and hence insufficient power of previous studies. The three variants are not associated with CAD related traits based on a GRASP database search (p<1x10^-4^). However, the overlap of the locus with immune cell histone marks suggest a link to inflammation. The lead SNP (rs12459996) of the TGFB1 locus is reported to increase the expression of the gene, which matches with the direction of effect reported for GA. In addition, the risk allele of the sub-locus rs1056854 is also associated with increased expression of the gene. The identified TGFB1 locus is also associated with expression of other genes (CCDC97, HNRNPUL1, AXL, BCKDHA) (see S2 Table). Because our study focused on genes that interact with GA, we did not discuss these genes at this locus. It is however possible, that these genes influence the risk of CAD as well. Indeed, several studies have demonstrated that a regulatory SNPs have effects on more than one gene. In a previous study, we found multiple genes per locus where either all SNPs, a subset or only one SNP increase the risk of CAD[35].

The molecular effects of TGFB have been extensively studied *in vitro* and *in vivo* models linking the gene to CAD risk. For example, TGFB1 has been associated with several CAD related phenotypes such as thrombosis, inflammation, hypertension and neointima growth[36–44]. However, the net effect of TGFB may vary. Indeed, TGFB1 may either increase or decrease inflammation, activate or deactivate macrophages, depending on the local cytokine environment[29]. This is mainly due to the fact that it acts through several signaling pathways affecting several CAD related phenotypes with partially opposing effect on risk [29, 33]. In early stages of the disease, TGFB1 may be atheroprotective and higher levels of TGFB1 have been reported to decrease the risk of atherosclerosis[29]. In fact, TGFB1 displayed dosage effects where lower levels of TGFB1 increased proliferation whereas higher levels inhibited proliferation in endothelial cells[36]. Vice versa, in the presence of disease increased TGFB1 signaling has been associated with increased restenosis by increasing neointima growth[37, 38]. Moreover, TGFB1 has been linked to accelerated thrombus formation by inducing platelet aggregation [39, 40] and the expression is increased in rats with traumatic deep vein thrombosis versus control rats [41]. TGFB1 was also found to inhibit nitric oxide in vascular endothelial cells linking higher levels of TGFB1 to increased blood pressure[42, 43]. In addition, TGFB1 expression was also reported to be increased in patients with hypertension[44].

Our study design has several limitations. Our findings are based on associations rather than on functional testing. We thus cannot infer the precise pathway that is affected by the genetic variants at the TGFB1 locus. However, functional effects of the risk allele on expression levels have been previously reported and go in the same direction of TGFB1 expression as the effects reported in the literature for GA. Moreover, the association between the TGFB1 locus and CAD risk reaches genome-wide significance, which can be regarded as a conclusive and scientifically important observation independently of the overlap with GA side effects including enhanced TGFB1 expression. Indeed, the links reported here, first between GA and CAD risk (drugs.com), second between GA and TGFB1[2] and third the genome-wide significant association of TGFB1 SNPs with CAD are each highly conclusive. Together they allow hypothesizing that TGFB1 is involved in the increased risk of CAD under GA treatment.

Taken together, our results imply mechanistic similarities between pharmacologic responses to GA treatment and genetic variants affecting CAD risk. While GA treatment is known to enhance TGFB1 expression and CAD risk, we here associate SNPs within the TGFB1 locus linked with CAD risk and enhanced TGFB1 expression. Thus, both the newly identified CAD risk alleles and GA appear to induce the expression of TGFB1, suggesting that the CAD risk alleles and the drug have similar effects on the gene product and subsequently on CAD risk. With an additive effect, the CAD risk alleles might also explain variable CAD risk under GA treatment. Finally, in this study, we identified TGFB1 as a new genome-wide significant locus affecting CAD risk.

## Supporting information

S1 Fig CAD association results. Association sub-loci signal for the genes reported to interact with glatiramer acetate. A) TGFB1: Transforming Growth Factor, Beta 1; B) IFNAR1: Interferon (Alpha, Beta And Omega) Receptor 1; C) CCR5: Chemokine (C-C Motif) Receptor 5 (Gene/Pseudogene); D) HLA-DRB1: Major Histocompatibility Complex, Class II, DR Beta 1.
(TIFF)

S2 Fig Association sub-loci signal for the TGFB1 locus. Independent sub-loci signal for the TGFB1 locus. A) rs12459996, B) rs1056854, C) rs75041078.
(TIFF)

S1 Table Additional samples for meta-analysis. Additional 21,934 CAD cases and 76.087 controls used for validation and extended meta-analysis. The sample size differs between SNPs for replication as not all SNPs are found in all studies.
(DOCX)

S2 Table Haploreg V4.1 annotation. Functional implication of the SNPs annotated with HaploReg version 4.1 database.
(XLSX)
