# Supplementary figures and images for: Genomic correlates of glatiramer acetate adverse cardiovascular effects lead to a novel locus mediating coronary risk
Source: PLoS One. 2017 Aug 22;12(8):e0182999. doi: 10.1371/journal.pone.0182999 (PMC5567477; doi:10.1371/journal.pone.0182999)

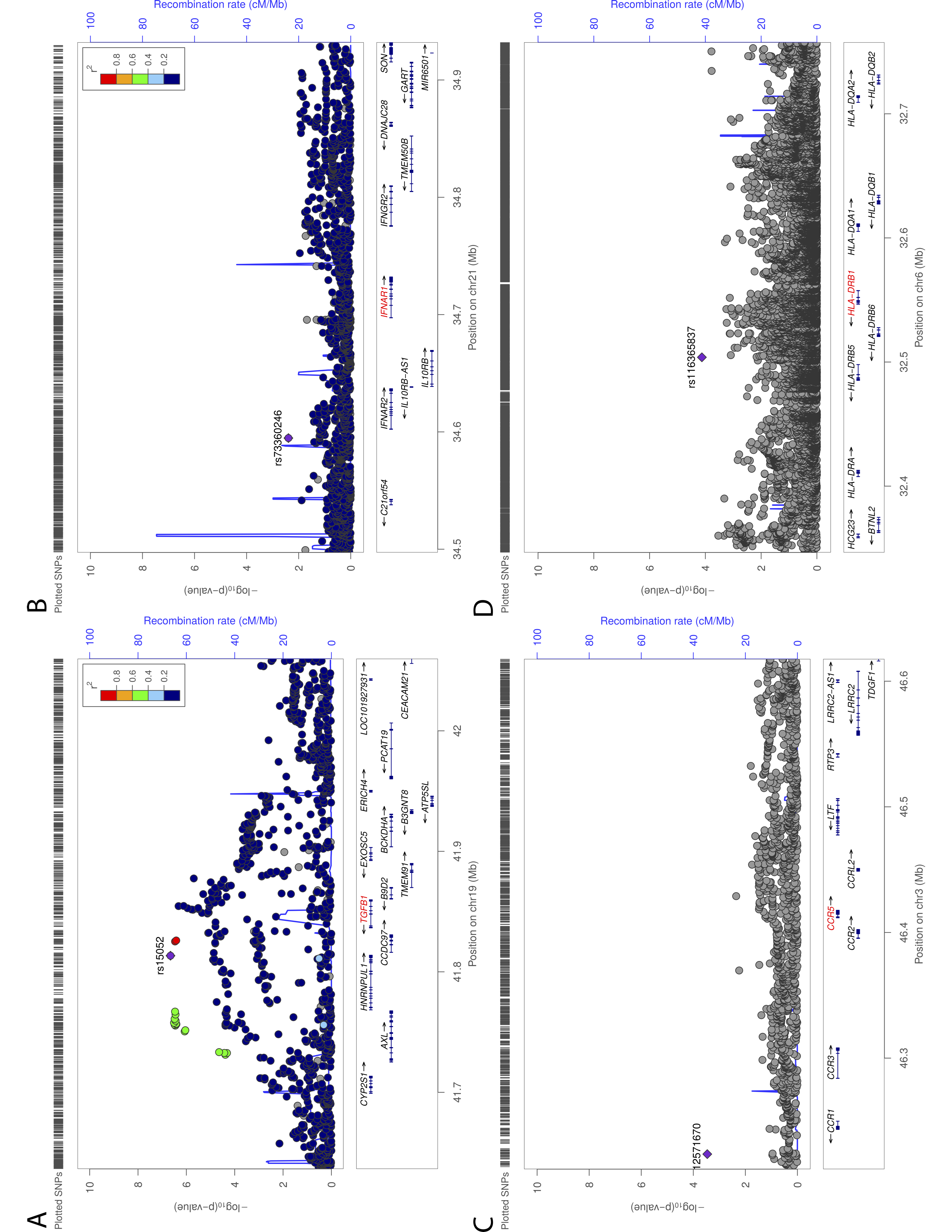

Supplement: S1 Fig — Association sub-loci signal for the genes reported to interact with glatiramer acetate. A) TGFB1: Transforming Growth Factor, Beta 1; B) IFNAR1: Interferon (Alpha, Beta And Omega) Receptor 1; C) CCR5: Chemokine (C-C Motif) Receptor 5 (Gene/Pseudogene); D) HLA-DRB1: Major Histocompatibility Complex, Class II, DR Beta 1. (TIFF) [file pone.0182999.s001.tiff]

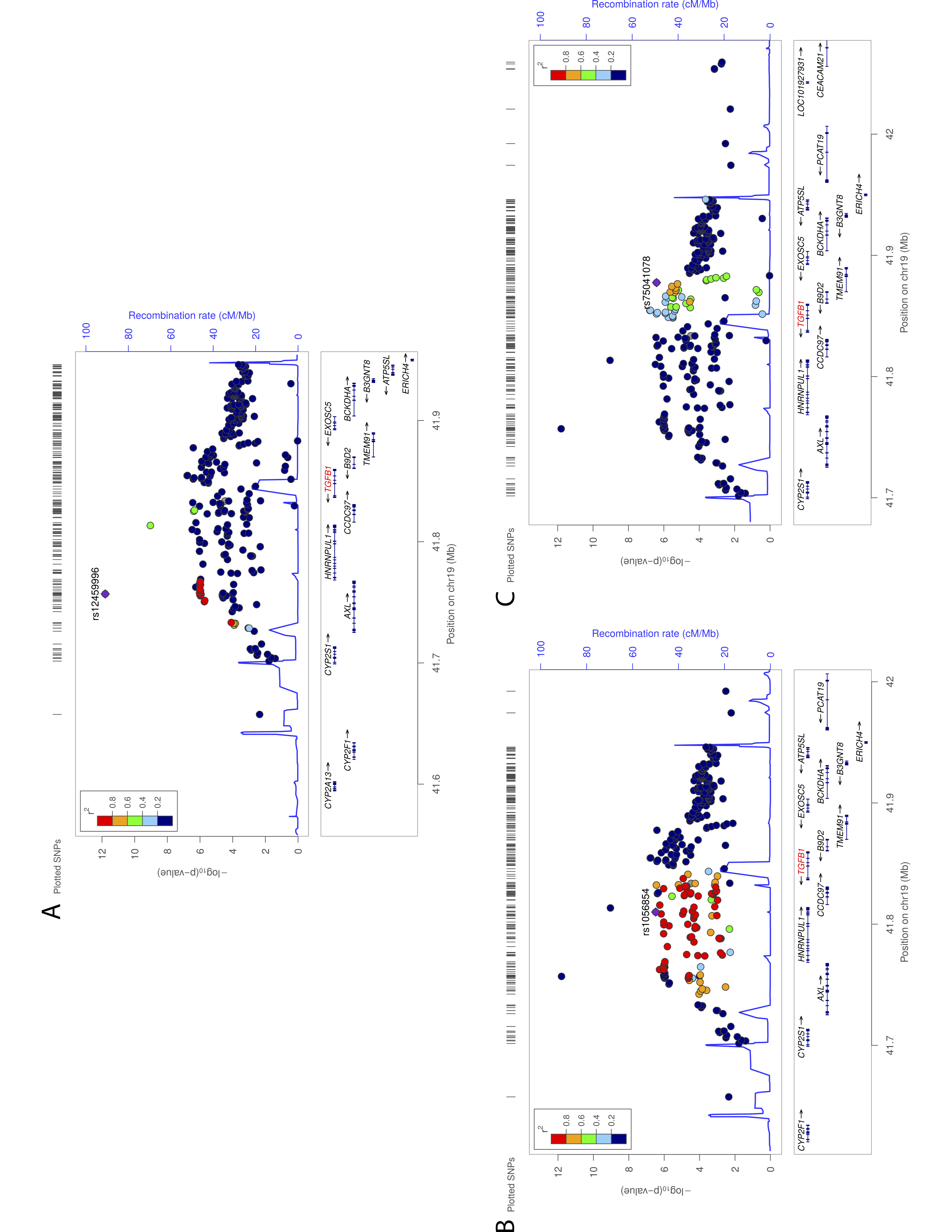

Supplement: S2 Fig — Independent sub-loci signal for the TGFB1 locus. A) rs12459996, B) rs1056854, C) rs75041078. (TIFF) [file pone.0182999.s002.tiff]
