# Supplementary material for: Genomic correlates of glatiramer acetate adverse cardiovascular effects lead to a novel locus mediating coronary risk
Source: PLoS One. 2017 Aug 22;12(8):e0182999. doi: 10.1371/journal.pone.0182999 (PMC5567477; doi:10.1371/journal.pone.0182999)
Supplement: S1 Table — Additional 21,934 CAD cases and 76.087 controls used for validation and extended meta-analysis. The sample size differs between SNPs for replication as not all SNPs are found in all studies. (DOCX) [file pone.0182999.s003.docx]

# Supplementary Table 1

| **Study** | **Phenotype** | **Full name** | **Sample Size Cases/controls** | **M(SD) Age** | **Female (%)**  **Cases/controls** | **Ref** |
| --- | --- | --- | --- | --- | --- | --- |
| ***CARDIoGRAM*** |  |  |  |  |  | PMID: 21378990 |
| CHARGE | CAD/MI | Cohorts for Heart and Aging Research in Genomic Epidemiology | 2287/22024 | 60.0(7.9)/ 63.1(8.0) | 33.4/ 59.6 | PMID: 23549178 |
| deCODE CAD | CAD/MI |  | 6640/27611 | 74.8 (11.8)/ 53.7(21.5) | 36.3/ 61.9 | PMID:17478679 |
| ***CARDIOoGRAMplus C4D MetaboChip*** |  |  |  |  |  | PMID: 23202125 |
| DILGOM | CAD/MI | The Dietary, Lifestyle, and Genetic determinants of Obesity and Metabolic syndrome study | 147/3844 | 56.6(9.5)/ 51.7(13.6) | 54.4 | PMID: 21179014 |
| EPIC | CAD/MI | The European Prospective Investigation into Cancer | 1526/2409 | 71.8(8.18)/ 60.3(9.3) | 47.4 | PMID: 23202125 |
| FRISC II - GLACIER | CAD/MI | Fragmin and Fast Revascularization during Instability in Coronary Artery Disease Gene x Lifestyle interactions And Complex traits Involved in Elevated disease Risk | 2937/6310 | 66.2(9.8)/ 50.5(9.3) | 49.8 | PMID: 20870969  PIMD: 10475181 |
| METISM | CAD/MI | METabolic Syndrome In Men | 224/1895 | 64.6(6.3)/ 58.7(7.3) | 0 | PMID:19223598 |
| MORGAM FIN | CAD | \| | 1242/1242 | 64.5(7.3)/ 60.9(7.8) | 14.7 | PMID: 15561751 |
| MORGAM FRA | CAD/MI | MONICA, Risk, | 183/183 | 57.6(3.0)/ 56.3(2.7) | 0 | PMID: 15561751 |
| MORGAM GER | CAD/MI | Genetics, Archiving, | 215/215 | 64.5(7.8)/ 58.9(8.2) | 20.9 | PMID: 15561751 |
| MORGAM ITA | CAD/MI | and Monograph | 151/151 | 61.3(9.3)/ 55.5(8.1) | 19.9 | PMID: 15561751 |
| MORGAM UNK | CAD/MI | \| | 164/164 | 59.7(4.1)/ 56.1(3.0) | 0 | PMID: 15561751 |
| PMB | CAD/MI | Pfizer-MGH-Broad | 922/4459 | 59.7(10.8)/ 57.6(10.3) | 45.7 | PMID: 23202125 |
| PopGen | CAD |  | 865/971 | 53.5(5.7)/ 54.7(15.2) | 30.5 | PMID: 18362232 |
| SCARF SHEEP | CAD/MI |  | 1525/1892 | 57.6(7.3)/ 50.5(7.0) | 28 | PMID: 23202125 |
| STR | CAD | Swedish Twin Registry | 447/1272 | 78.9(9.7)/ 73.1(11.0) | 55.8 | PMID: 8981957 |
| ***addional 1000G*** |  |  |  |  |  |  |
| GerMIFS V | MI | German Myocardial Infarction Family Studies | 2459/1445 |  | 24.2/ 52.6 | - |

Additional 21,934 CAD cases and 76.087 controls used for validation and extended meta-analysis. The sample size differs between SNPs for replication as not all SNPs are found in all studies.
